# Study on influencing factors of anthracycline-induced subclinical cardiotoxicity in DLBCL patients administered (R)-CHOP

**DOI:** 10.1186/s12885-022-10085-6

**Published:** 2022-09-17

**Authors:** Qian Dong, Wenxin Ou, Mei Wang, Tiantian Jiang, Yue Weng, Xi Zhou, Xiaoqiong Tang

**Affiliations:** 1 Department of Cardiology, The First Affiliated Hospital of Chongqing Medical University, Yuzhong, Chongqing, China; 2 Department of Hematology, The First Affiliated Hospital of Chongqing Medical University, Yuzhong, Chongqing, China

**Keywords:** Diffuse large B-cell lymphoma, Cardiotoxicity, Anthracycline, Influencing factor

## Abstract

**Background:**

Anthracycline-induced cardiotoxicity is an irreversible cardiac cell injury. Therefore, it’s very important to identify influencing factors of anthracycline-induced subclinical cardiotoxicity (AISC). This study was designed to analyze the influencing factors of AISC in patients with diffuse large B-cell lymphoma (DLBCL) treated with the (R)-CHOP chemotherapy regimen.

**Methods:**

This is an ongoing observational prospective clinical trial. All patients underwent conventional echocardiography and speckle tracking echocardiography at the time of enrollment and during treatment. Changes of global longitudinal peak systolic strain were assessed after 3 cycles of (R)-CHOP chemotherapy, and patients were divided into the AISC and No-AISC groups. Demographic data, clinical variables, and biochemical variables were measured. Regression models, receiver operating characteristic curve analysis, and difference values were used to explore the relationships between variables and AISC.

**Results:**

Among 70 patients who completed 3 cycles of (R)-CHOP chemotherapy, 26 developed AISC. In multiple logistic regression, HDL-C (*P* = 0.047), ApoA1 (*P* = 0.022), TG (*P* = 0.029) and e’ (*P* = 0.008) were associated with AISC. The combination of HDL-C and NT-proBNP had the highest area under curves (AUC) for the diagnosis of AISC than HDL-C and NT-proBNP alone (AUC = 0.752, 95%CI: 0.63–0.87, *P* = 0.001). Between the No-AISC and AISC groups, there was no significant difference in HDL-C, ApoA1, and e’ at baseline and after 3 cycles of chemotherapy, respectively. The dynamic changes of HDL-C, ApoA1, and e’ from baseline to the end of the 3^rd^ cycle of chemotherapy showed statistically significant differences.

**Conclusions:**

HDL-C, ApoA1, TG, and e’ are independent predictive factors in DLBCL cases treated with the (R)-CHOP chemotherapy regimen. The combination of HDL-C and NT-proBNP may improve the predictive ability for AISC in patients with DLBCL administered 3 cycles of (R)-CHOP chemotherapy. Dynamic changes of HDL-C, ApoA1, and e’ may be meaningful for predicting AISC.

**Trial registration:**

Our study was registered in the Chinese Clinical Trial Registry (Approval ID. ChiCTR2100054721 http://www.chictr.org.cn/showproj.aspx?proj=145082).

## Background

With the rapid development of cancer treatment in recent decades, the survival rate of patients diagnosed with solid and hematologic malignancies has largely increased [1]. Unfortunately, chemotherapy often causes acute or chronic cardiovascular complications, which are major causes of noncancer mortality among survivors [2, 3]. In the era of targeted therapy, anthracycline-containing therapy still plays a prominent role in non-Hodgkin’s lymphoma (NHL). Anthracyclines cause a variety of cardiotoxic effects, including electrocardiographic changes, arrhythmia, conduction abnormalities, and left ventricular dysfunction, and are one of the most commonly used groups of chemotherapeutic agents that induce cardiotoxicity [2, 4]. Anthracycline-induced cardiotoxicity is an irreversible cardiac cell injury [5, 6]. According to 2020 ESMO consensus recommendations and International Cardio-Oncology Society (IC-OS) consensus statement, there is a need for early intervention once anthracycline-induced cardiotoxicity is detected [7, 8], but only a small number of cardioprotective treatments have been tested in humans and currently, no clear guidelines or worldwide accepted therapies exist [5, 6]. Therefore, it seems very important to identify influencing factors of anthracycline-induced subclinical cardiotoxicity (AISC), which was defined as normal LVEF but a relative GLS decrease from baseline of ≥ 12% according to IC-OS consensus statement [8]. Patients with subclinical cardiotoxicity benefit from the discovery of influencing factors and early detection of pathophysiological changes that lead to clinical heart disease by providing an opportunity for timely intervention and prevention. Recent studies have identified cardiotoxicity at doxorubicin doses in the range of 100‐150 mg/m^2^ can cause cardiotoxicity [9]. This is much lower than previously recognized. Diffuse large B-cell lymphoma (DLBCL) is the most common subtype of NHL [10]. The (R)-CHOP chemotherapy regimen (rituximab, cyclophosphamide, doxorubicin, vincristine, and prednisone) has been the standard treatment option for DLBCL for decades [11]. The influencing factors of anthracycline-induced subclinical cardiotoxicity are not well investigated. This study was designed to analyze the influencing factors of AISC in patients with DLBCL treated with the (R)-CHOP chemotherapy regimen.

## Methods

### Study subjects

This is an ongoing clinical observational prospective trial. The main inclusion criteria were: consecutive chemotherapy-naïve patients diagnosed with DLBCL; planned treatment with the (R)-CHOP chemotherapy regimen; age ≥ 18 years and < 80 years; Eastern Cooperative Oncology Group (ECoG) score ≤ 2; left ventricular ejection fraction (LVEF) ≥ 50%. The main exclusion criteria were: unable to receive a full-dose (R)-CHOP, a history of myocarditis, myocardial ischemia, myocardial infarction, arrhythmia requiring medical intervention, and clinical or subclinical pericardial effusion, a history of other cancers, and severe active infections such as hepatitis, syphilis, or human immunodeficiency virus (HIV) infection.

Patients received full-dose (R)-CHOP (cyclophosphamide at 750 mg/m^2^ on D1, vincristine at 1.4 mg/m^2^ [maximum 2 mg] on D1, 100 mg prednisone on D1-5, and doxorubicin at 50 mg/m^2^ on D1, with or without rituximab at 375 mg/m^2^ on D1) in each cycle.

All patients underwent conventional 2D transthoracic echocardiography and 2D speckle tracking echocardiography (STE) at the time of enrollment (baseline), after 3 cycles of (R)-CHOP chemotherapy, after 6 cycles of (R)-CHOP chemotherapy, and during follow-up (6 and 12 months, respectively, after treatment completion). Left ventricular ejection fraction (LVEF), fractional shortening (FS), left ventricular diastolic dimension (LVDd), left ventricular mass index (LVMI), mitral inflow E velocity, mitral e’ velocity, E/e’, and global longitudinal peak systolic strain (GLS) were measured to assess left ventricular systolic dysfunction by the same ultrasound machine. Echocardiography was performed according to the recommendations of the Chinese Society of Echocardiography. Subclinical cardiotoxicity was defined as normal LVEF but a relative GLS decrease from baseline of ≥ 12% [(baseline – current GLS)/baseline GLS] according to International Cardio-Oncology Society (IC-OS) consensus statement [8]. Changes of GLS were assessed after 3 cycles of (R)-CHOP chemotherapy, and patients were divided into the AISC and No-AISC groups. The accumulated dose of doxorubicin was 150 mg/m^2^ after 3 cycles of (R)-CHOP chemotherapy.

Demographic data and clinical variables, including age, gender, body mass index (BMI), smoking history (an adult who has smoked at least 100 cigarettes in his or her lifetime), drinking history, diabetes, carotid plague, heart rate (HR), and hypertension, were collected. Biochemical variables, including high-density lipoprotein cholesterol (HDL-C), low-density lipoprotein cholesterol (LDL-C), apolipoprotein A1 (ApoA1), cardiac troponin T (cTnT), N-terminal prohormone of brain natriuretic peptide (NT-proBNP), white blood cell (WBC) count, red blood cell (RBC) count, hemoglobin (Hb), platelet (PLT), Urea, creatinine (Cr), uric acid (UA), total cholesterol (TC) and total triglyceride (TG) were measured at the time of enrollment (baseline), after 3 cycles of (R)-CHOP chemotherapy, after 6 cycles of (R)-CHOP chemotherapy, and during follow-up (6 and 12 months, respectively, after treatment completion).

The study was registered in Chinese Clinical Trial (Approval NO. ChiCTR2100054721). The study was conducted in accordance with the Declaration of Helsinki and approved by the ethics committee of the first affiliated hospital of Chongqing Medical University (Approval NO. 2018–016). All patients provided informed consent.

### Reproducibility

A total of 15 patients were randomly selected for the assessment of intra- and interobserver variability in GLS. Measurements were performed in the same datasets by one observer twice and verified by a blinded second reviewer. The results of the two measurements were compared by the t-test, and no statistically significant differences were found (*P* > 0.05).

### Statistical analysis

Continuous variables were presented as mean and standard deviation and compared by the t-test or ANOVA as appropriate. Non-normally distributed variables were presented as median (Q1- Q3) and compared by the Wilcoxon Mann–Whitney test or Kruskal Wallis test. Categorical variables were presented as n (%) and compared by the Chi-square or Fisher’s exact test as appropriate. A stepwise multiple logistic regression analyses with forward section were conducted to identify the influencing factors for AISC. Confounding factors were age, gender, and *P* < 0.10 (smoking history, BMI, NT-proBNP, e’, TG, LDL-C) in the simple logistic regression analysis (Table 2). The outcome variable was whether patients were detected with AISC by STE after 3 cycles of (R)-CHOP chemotherapy. Variables those with *P* ≥ 0.10 in the simple logistic regression analysis were added one at a time in the multiple model to access the effect of potential predictors on AISC in patients with DLBCL. The predictive ability of biomarkers measured at baseline for the prognosis of patients with AISC was estimated by receiver operating characteristic (ROC) curve analysis. *P* < 0.05 was considered statistically significant. IBM SPSS V.22.0 was used for statistical analysis.

## Results

### Study population and baseline characteristics

Patients newly diagnosed with DLBCL, planned for treatment with (R)-CHOP chemotherapy regimen, were enrolled in our trial. Among them, 70 patients completed 3 cycles of (R)-CHOP chemotherapy, and 26 patients developed AISC. Baseline patient characteristics in the AISC and No-AISC groups are listed in Table 1, in which age, gender and variables that were found statistically important (*P* < 0.20) are displayed. Variables analyzed but found not statistically important (heart rate, hypertension, diabetes, drinking history, carotid plague, LVEF, FS, LVDd, LVMi, E/e’, HDL-C, ApoA1, cTnT, WBC count, RBC count, Hb, PLT, Urea, Cr, UA, ALT and AST, *P* ≥ 0.2) are not displayed in Table 1.

**Table 1:** Baseline characteristics of DLBCL patients completed 3 cycles of (R)-CHOP chemotherapy

	All patients	No-AISC	AISC	*P*-value
	*N* = 70	*N* = 44	*N* = 26	
Age (year)	55.47 ± 13.63	55.50 ± 14.86	55.42 ± 11.54	0.982
Male/female (n)	33/37	20/24	13/13	0.713
BMI (kg/m^2^)	22.85 ± 3.31	22.12 ± 3.20	24.10 ± 3.18	**0.014**
Smoking(%)	17 (24.29)	7 (15.91)	10 (38.46)	**0.033**
GLS (-%)	20.04 ± 2.24	19.73 ± 2.29	20.58 ± 2.08	0.125
E (cm/s)	67.79 ± 13.01	66.01 ± 11.47	70.81 ± 15.01	0.137
e’(cm/s)	7.27 ± 2.03	6.92 ± 1.98	7.85 ± 2.02	0.067
LDL-C (mmol/L)	2.56 ± 0.77	2.42 ± 0.68	2.80 ± 0.85	**0.046**
NT-proBNP (ng/L)	54.00 (31.00, 127.00)	70.50 (32.25, 132.25)	41.50 (24.25, 76.50)	0.070
TC (mmol/L)	4.19 ± 0.93	4.07 ± 0.86	4.39 ± 1.02	0.168
TG (mmol/L)	1.36 (1.02, 1.68)	1.25 (0.91, 1.55)	1.54 (1.27, 1.92)	**0.009**

Values are expressed as mean ± standard deviation, n (%), or median (Q1-Q3). Bold values indicate statistical significance (*P* < 0.05)

*AISC* Anthracycline-induced subclinical cardiotoxicity, *BMI* Body mass index, *DLBCL* Diffuse large-B cell lymphoma, *E* mitral inflow E velocity, *e’* mitral e’ velocity, *GLS* Global longitudinal peak systolic strain, *LDL-C* Low-density lipoprotein cholesterol, *NT-proBNP* N terminal-pro brain natriuretic peptide, *TC* Total triglyceride, *TG* Total cholesterol

### Logistic regression analysis of baseline characteristics and anthracycline-induced subclinical cardiotoxicity after 3 cycles of (R)-CHOP chemotherapy in DLBCL patients

BMI (OR = 1.219, 95%CI 1.033–1.438, *P* = 0.019), smoking history (OR = 3.304, 95%CI 1.067–10.226, *P* = 0.038) were significantly associated with AISC in DLBCL patients (Table 2). Variables analyzed but found not statistically important (heart rate, hypertension, diabetes, drinking history, carotid plague, LVEF, FS, LVDd, LVMi, E/e’, ApoA1, cTnT, WBC count, RBC count, Hb, PLT, Urea, Cr, UA, TC, ALT and AST, *P* ≥ 0.2) are not displayed in Table 2.

**Table 2:** Effects of various variables on anthracycline-induced subclinical cardiotoxicity in DLBCL patients completed 3 cycles chemotherapy

Characteristics	OR (95%CI)	*P*-value
Age, per 1 year	1.000 (0.964, 1.036)	0.982
Male, yes versus no	0.833 (0.316, 2.201)	0.713
BMI, per 1 kg/m^2^	1.219 (1.033,1.438)	**0.019**
Smoking, yes versus no	3.304 (1.067, 10.226)	**0.038**
GLS, per -1%	1.200 (0.949, 1.517)	0.129
E, per 1 cm/s	1.030 (0.991, 1.070)	0.138
e’, per 1 cm/s	1.258 (0.980, 1.614)	0.071
LDL-C, per 1 mmol/L	2.016 (0.992,4.095)	0.053
HDL-C, per 1 mmol/L	0.371 (0.082, 1.683)	0.199
NT-proBNP, per 1 ng/L	0.993 (0.986,1.000)	0.066
TG, per 1 mmol/L	1.806 (0.942,3.462)	0.075

Bold values indicate statistical significance

*BMI* Body mass index, *cTnT* cardiac troponin T, *Cr* Creatinine, *FS* Fractional shortening, *GLS* Global longitudinal peak systolic strain, *HDL-C* High-density lipoprotein cholesterol, *LDL-C* Low-density lipoprotein cholesterol, *NT-proBNP* N terminal-pro brain natriuretic peptide, *TG* Total cholesterol

A stepwise multiple regression analysis with the forward selection method was used to access the effect of potential predictors on AISC in patients with DLBCL. Results showed that e’, ApoA1, HDL-C, and TG significantly influenced the risk of AISC after 3 cycles of (R)-CHOP chemotherapy (Table 3).

**Table 3:** Multiple logistic regression analysis on anthracycline-induced subclinical cardiotoxicity in DLBCL patients completed 3 cycles chemotherapy

Characteristics	OR (95%CI) (Adjusted)	*P*-value
Age, per 1 year	1.053 (0.987, 1.124)	0.118
Male, yes versus no	3.212 (0.680, 15.185)	0.141
Smoking, yes versus no	2.601 (0.450, 15.016)	0.285
BMI, per 1 kg/m^2^	1.239 (0.967, 1.589)	0.090
NT-proBNP, per 1 ng/L	0.995 (0.988, 1.002)	0.179
e’, per 1 cm/s	2.045 (1.201, 3.481)	**0.008**
TG, per 1 mmol/L	2.810 (1.114, 7.088)	**0.029**
LDL-C, per 1 mmol/L	0.833 (0.315, 2.200)	0.712
ApoA1, per 1 g/L	0.032 (0.002, 0.611)	**0.022**
HDL-C, per 1 mmol/L	0.057 (0.003, 0.964)	**0.047**

Age, gender, and variables with *P* < 0.10 in Table 2 (smoking history, BMI, NT-proBNP, e’, TG and LDL-C) were considered as potential cofounding factors

Bold values indicate statistical significance (*P* < 0.05)

*ApoA1* Apolipoprotein A1, *BMI* Body mass index, *DLBCL* Diffuse large-B cell lymphoma, *e’* mitral e’ velocity, *HDL-C* High-density lipoprotein cholesterol, *LDL-C* Low-density lipoprotein cholesterol, *NT-proBNP* N terminal-pro brain natriuretic peptide, *TG* Total triglyceride, *OR* Odds ratio

### Prognostic implication comparison between influencing factors in DLBCL patients with 3 cycles of (R)-CHOP chemotherapy

**Fig. 1:**
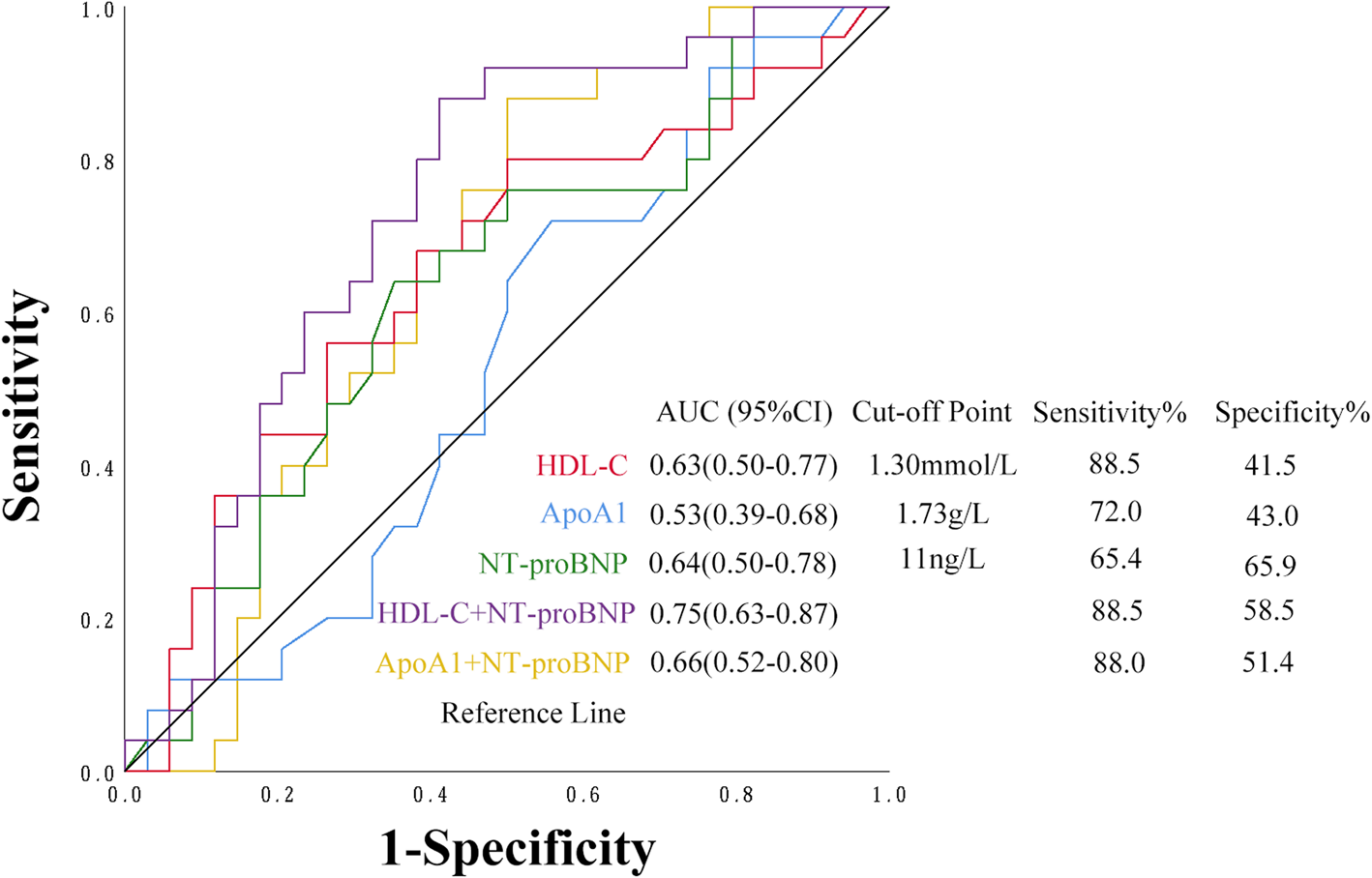
ROC curve analysis of the predictive values of HDL-C, ApoA1, NT-proBNP and their combination. AISC: anthracycline-induced subclinical cardiotoxicity; AUC: areas under the curve; ApoA1: apolipoprotein A1; HDL-C: high-density lipoprotein cholesterol; NT-proBNP: N terminal-pro brain natriuretic peptide; ROC: receiver operating characteristic

We analyzed the predictive ability of biomarkers measured at baseline for the prognosis of AISC. In DLBCL patients who completed 3 cycles of (R)-CHOP chemotherapy, HDL-C was not better than NT-proBNP in predicting AISC, but their combined use provided an elevated prognostic value (area under curve [AUC] = 0.752, *P* = 0.001 vs. HDL-C; AUC = 0.631, *P* = 0.068 vs. NT-proBNP; AUC = 0.640, *P* = 0.054). ROC curve analysis of AISC demonstrated that HDL-C was best detected with a cut-off point of 1.30 mmol/L (sensitivity of 88.50% and specificity of 41.50%), with an AUC of 0.631 (95% CI 0.497–0.765). NT-proBNP was best detected with a cut-off point of 11 ng/L (sensitivity of 65.40% and specificity of 65.90%), with an AUC of 0.640 (95%CI 0.504–0.777). The sensitivity of their combination was 88.50%, and specificity was 58.50%, with an AUC of 0.752 (95%CI 0.634–0.871). ApoA1 was not better than HDL-C in predicting AISC (AUC = 0.530, *P* = 0.776 vs. AUC = 0.631, *P* = 0.068). And ApoA1 combined with NT-proBNP was not better than HDL-C combined with NT-proBNP in predicting AISC (AUC = 0.660, *P* = 0.025 vs. AUC = 0.752, *P* = 0.001) (Fig. 1). The difference value of e’ had a predictive capability of AISC with a sensitivity of 64.5%, specificity of 68.4%, and AUC = 0.73 (95%CI:0.60–0.85, *P* = 0.002). TG showed no benefit in predicting AISC (AUC = 0.31).

### Significance of dynamic changes of influencing factors in DLBCL patients at baseline and after 3 cycles of (R)-CHOP chemotherapy

Between the No-AISC and AISC groups, there was no significant difference in HDL-C, ApoA1 and e’ at baseline and after 3 cycles of chemotherapy, respectively. TG in patients with AISC was higher than without, no matter at baseline or after 3 cycles of chemotherapy (Table 4).

**Table 4:** Dynamic changes of variables between baseline and after 3 cycles of (R)-CHOP in DLBCL patients

Variable	At baseline	After 3 cycles of (R)-CHOP	*P*-value	d (after—baseline)^a^
HDL-C				
No-AISC	1.07 (0.81, 1.38)	1.27 (0.96, 1.49)	**0.010**	0.18 (-0.14, 0.27)
AISC	0.97 (0.86, 1.20)	1.08 (0.81, 1.61)	0.219	0.09 (-0.16, 0.28)
*P*-value	0.410	0.062		**0.005**
ApoA1				
No-AISC	1.18 ± 0.36	1.34 ± 0.2	**0.038**	1.22 ± 0.33
AISC	1.17 ± 0.29	1.24 ± 0.30	0.145	1.10 ± 0.30
*P*-value	0.913	0.162		0.795
e’				
No-AISC	6.92 ± 1.98	7.04 ± 2.42	0.599	0.14 ± 1.65
AISC	7.85 ± 2.02	6.52 ± 2.08	**0.001**	-1.33 ± 0.34
*P*-value	0.067	0.365		**0.001**
TG				
No-AISC	1.25 (0.91, 1.55)	1.38 (0.98, 2.00)	**0.004**	0.22 (-0.08, 0.69)
AISC	1.54 (1.27, 1.92)	2.01 (1.53, 3.15)	0.081	0.28 (-0.23, 1.23)
*P*-value	**0.009**	**0.003**		0.814

Values are expressed as mean ± standard deviation or median (Q1-Q3). Bold values indicate statistical significance

^a^ Difference value of variables measured after 3 cycles of (R)-CHOP and at baseline

*AISC* Anthracycline-induced subclinical cardiotoxicity, *ApoA1* Apolipoprotein A1, *DLBCL* Diffuse large-B cell lymphoma, *HDL-C* High-density lipoprotein cholesterol, *TG* Total triglyceride

In patients without AISC, the dynamic changes of HDL-C and ApoA1 from baseline to the end of the 3^rd^ cycle of chemotherapy both elevated, showing statistically significant differences (*P* = 0.010 and *P* = 0.038). The difference value of HDL-C measured at the end of the 3^rd^ cycle of chemotherapy and at baseline had significant difference (*P* = 0.005) between patients with and without AISC. It is clear that the value of e’ decreased significantly from baseline to the end of the 3^rd^ cycle of chemotherapy in patients with ASIC (d = -1.33 ± 0.34), while that of e’ had an increasing trend in patient without AISC (d = 0.14 ± 1.65). Between the two groups, the difference value of e’ measured at the end of the 3^rd^ cycle and at baseline saw a statistically significant difference (*P* = 0.001).

## Discussion

We conducted a clinical observational trial and found that HDL-C, ApoA1, TG, and e’ were independent influencing factors of anthracycline-induced subclinical cardiotoxicity (AISC) in diffuse large B-cell lymphoma (DLBCL) patients receiving 3 cycles of (R)-CHOP. Besides, the combination of baseline HDL-C and baseline NT-proBNP was a feasible predictive index for AISC. Among patients who were not detected to have AISC after 3 cycles of chemotherapy, the level of HDL-C and ApoA1 both saw significant increases, as expected. Among patients detected to have AISC after 3 cycles of chemotherapy, the value of e’ saw a significant decrease in accordance with the theory that e’ is positively correlated with cardiac function. Therefore, paying attention to dynamic changes of HDL-C, ApoA1, and e’ may be helpful for predicting AISC.

There is currently no proven treatment that reverses cardiotoxicity induced by chemotherapy. Detecting subclinical cardiotoxicity provides clinicians and patients an opportunity for early intervention and prevention of symptomatic cardiotoxicity. Thus, there is a need to identify, test, and validate biomarkers that predict early heart damage in cancer patients receiving cardiotoxic agents. NT-proBNP is a quantitative marker of HF and provides the most accurate noninvasive tool for estimating intracardiac filling pressures and end-diastolic wall stress [12]. Increased NT-proBNP can reflect early cardiotoxicity, but mostly in clinical cardiotoxicity when cardiomyocyte damage has already occurred [13]. Serum troponins are sensitive and specific biomarkers for evaluating ischemic heart damage or myocardial infarction in the clinical setting [14]. Early elevation of serum cTnT during anthracycline treatment is associated with increased long-term anthracycline-induced cardiotoxicity [15]. NT-proBNP and cTnT showed no statistically significant influences on AISC in our study, and we found that HDL-C, ApoA1, TG, and e’ were influencing factors of AISC. This might indicate that serum lipid management can be an early target for protecting against AISC. Mitral e’ velocity might be an alternative indicator of AISC, which is easier and cheaper to be measured than GLS. HDL-C is a highly effective biomarker for predicting cardiovascular risk and its use for this purpose is undisputed. Many prospective studies from different racial and ethnic groups worldwide have confirmed that HDL-C is a strong, consistent, and independent predictor of incident cardiovascular events such as myocardial infarction and ischemic stroke [16, 17]. ApoA1 is the most abundant protein in HDL and is linked to many beneficial effects of HDL [18], and it is reported that HDL quality is highly dependent on the abundance and function of ApoA1 [19]. ApoA1 constitutes approximately 70% of HDL proteins and is present on virtually all HDL particles and is reported to determine the function of HDL [20]. However, whether ApoA1 has a better predictive ability than HDL-C is still controversial [21, 22]. We used ROC curve analysis to estimate the predictive efficacy of HDL-C, ApoA1, and NT-proBNP. Our data showed that the combination of HDL-C and NT-proBNP may improve the predictive ability for AISC than HDL-C and NT-proBNP alone. ApoA1 was not better than HDL-C, and ApoA1 combined with NT-proBNP was not better than HDL-C combined with NT-proBNP in predicting AISC in patients with DLBCL administered 3 cycles of (R)-CHOP chemotherapy. In addition, there were no significant differences between the No-AISC and AISC groups in HDL-C, ApoA1, and e’ at baseline and after 3 cycles of chemotherapy, respectively. However, their dynamic changes showed statistically significant differences. These results confirmed that observing the dynamic changes of HDL-C, ApoA1, and e’ may be a new way to early detect AISC.

Our study found that patients who had AISC after 3 cycles of anthracycline-contained chemotherapy had higher TG, lower HDL-C, and lower ApoA1 at baseline. Dyslipidemia is a shared risk factor for cardiovascular disease risk and anthracycline-induced cardiotoxicity [23], and dyslipidemia interacting with cancer therapies can increase the risk of cardiac disease [24], thus it is important to early control serum lipids. Fibrates are the best drugs for reducing triglyceride levels, lower by 50% or more in many patients and increase HDL-C levels by 15% [25]. Ezetimibe, niacin, omega-3 fatty acids, and microsomal triglyceride transport protein inhibitor are also drugs for reducing triglyceride levels [25]. Preclinical studies have found that HDL and its precursor protein ApoA1 can protect against anthracycline-induced cardiotoxicity [26–28]. From above studies, it is possible that lipid management may be taken to tackle cardiotoxic problems in the future.

Anthracycline-induced cardiotoxicity is an irreversible cardiac cell injury [5, 6] which influences patients’ quality of life and survive. Therefore, identifying influencing factors and predictive markers of AISC as well as intervention targets are particularly important. Our results provided evidence and hint for future cardiac protection.

### Limitation

There were several limitations in the present study. Firstly, we reported a single-center, medium-sample data with inherent bias. A multi-center, large-sample study is needed to verify these findings. Secondly, a case–control study design was used in our study, and prospective cohort studies are needed to verify the results. Thirdly, the data of later cycles of chemotherapy were not comprehensively analyzed because of the small size of the sample, so our further research needs to pay more attention to this aspect.

## Conclusions

In summary, baseline HDL-C, ApoA1, TG and e’ are independent influencing factors for AISC in DLBCL cases treated with the (R)-CHOP chemotherapy regimen. The combination of baseline HDL-C and baseline NT-proBNP shows satisfactory predictive ability for AISC in patients with DLBCL administered 3 cycles of (R)-CHOP chemotherapy. Dynamic changes of HDL-C, ApoA1 and e’ may be meaningful for predicting AISC.

## Data Availability

The data that support the findings of this study are available from the Chinese Clinical Trial Registry (http://www.chictr.org.cn/showproj.aspx?proj=145082) but restrictions apply to the availability of these data, which were used under license for the current study, and so are not publicly available. Data are however available from the corresponding author upon reasonable request.

## Abbreviations

AISC: Anthracycline-induced subclinical cardiotoxicity
AST: Aspartate transaminase
ApoA1: Apolipoprotein A1
ALT: Alanine aminotransferase
BMI: Body mass index
cTnT: Cardiac troponin T
Cr: Creatinine
DLBCL: Diffuse large-B cell lymphoma
E: Mitral inflow E velocity
e’: Mitral e’ velocity
FS: Fractional shortening
GLS: Global longitudinal peak systolic strain
Hb: Hemoglobin
HDL-C: High-density lipoprotein cholesterol
LVEF: Left ventricular ejection fraction
LVDd: Left ventricular diastolic dimension
LVMi: Left ventricular mass index
LDL-C: Low-density lipoprotein cholesterol
NT-proBNP: N terminal-pro brain natriuretic peptide
PLT: Platelet
RBC: Red blood cell
TC: Total triglyceride
TG: Total cholesterol
UA: Uric acid
WBC: White blood cell
